# Differential Predation by Age and Sex Classes in Blue Wildebeest in Serengeti: Study of a Modern Carnivore Den in Olduvai Gorge (Tanzania)

**DOI:** 10.1371/journal.pone.0125944

**Published:** 2015-05-27

**Authors:** Mari Carmen Arriaza, Manuel Domínguez-Rodrigo, Cayetana Martínez-Maza, Audax Mabulla, Enrique Baquedano

**Affiliations:** 1 Departamento de Geología, Geografía y Medio Ambiente, Universidad de Alcalá, Edificio de Ciencias, Campus Externo, Ctra. A-II-km 33,600 C. P. 28871 Alcalá de Henares, Madrid, Spain; 2 Instituto de Evolución en África (IDEA), Madrid, Spain; 3 Departamento de Prehistoria, Universidad Complutense, Madrid, Spain; 4 Departamento de Paleobiología, Museo Nacional de Ciencias Naturales, Madrid, Spain; 5 Department of Archaeology, University of Dar es Salaam, Dar es Salaam, Tanzania; 6 Museo Arqueológico Regional de la Comunidad de Madrid, Alcalá de Henares, Madrid, Spain; University of Sydney, AUSTRALIA

## Abstract

Age and sex selection of prey is an aspect of predator ecology which has been extensively studied in both temperate and African ecosystems. This dimension, along with fecundity, survival rates of prey and mortality factors other than predation are important in laying down the population dynamics of prey and have important implications in the management of species. A carnivore den located in the short-grassland ecological unit of the Serengeti was studied. Sex- and age- class (using five age categories) of the wildebeest remains recovered were analyzed through horn morphology, biometrics of the bones and tooth wear patterns. We compared our results with previous studies from lion and hyaena kills through multivariate analyses. Seasonality of the accumulation was analyzed through tooth histology. PCA and CVA results show that age class selection by predators depends on season, habitat-type, and growth rate of the wildebeest population. Female-biased predation was found to contradict classical hypotheses based on territorial male behaviour. The lion and spotted hyaena showed strong selection on age classes, contrary to previous studies. Migratory wildebeest sex ratio is regulated through differential predation by seasons and female deaths in the wet season are a trade-off for population stability. These data are crucial for an effective management of the species and the new method created may be useful for different carnivore species and their prey.

## Introduction

Age and sex selection of prey is an aspect of predator ecology which has been extensively studied (cf. [1–4]). This aspect along with fecundity and survival rates of prey and factors affecting mortality other than predation, are important to lay down the population dynamics of prey and have important implications in the management of species [5–6]. These data are difficult to measure and ecosystems are dynamic, making it difficult to extrapolate from one area to another, or even from one time period to another within the same area [7].

One of the most extensively studied ungulate species in Serengeti National Park (SNP) is the blue wildebeest (*Connochaetes taurinus* Burchell) [5,8–14]. The two main predators of wildebeest in the Serengeti are the lion (*Panthera leo* Linnaeus) and the spotted hyaena (*Crocuta crocuta* Erxleben) [15–16]. Traditionally, it has been argued that cursorial predators like spotted hyenas show attritional age-class profiles, while ambush predators like the lion show catastrophic age-class profiles [4,17]. Although both predators hunt wildebeest in very different ways, they are very similar in targeting healthy animals, but differ in their age profiles: lion kills are younger than those of hyenas [16] for adult wildebeests. Ungulate territorial behavior could contribute to a higher male mortality [18–19]. Different hypotheses such as “post-rutting reproductive cost” [12], “satellite male-hypothesis” [16] or “solitary bull hypothesis” attempt to account for the fact that male ungulates are more vulnerable to predation [5]. Both hyenas and lions have been reported to show male-biased wildebeest differential predation; lions possibly because ungulate males are often solitary and hyenas perhaps because there may be proportionately more sick males in the wildebeest population [2,4].

Differential wildebeest age and sex classes selected by predators have frequently been reported, but the results sometimes seem not to be consistent with the dynamics of the population. Sinclair [12] pointed out that, if the wildebeest sex ratio is 1:1 in the Serengeti and predation is male-biased, another, as yet unknown, mechanism will regulate female mortality. Mduma [5] suggested that differential predation hypotheses perhaps do not take in account the possibility that the population age structure could determine the probability of predation. Mduma [5] also found that hyenas hunt animals with poorer health than those studied by Sinclair and Arcese [16]. Our study targets addressing these interpretations by analyzing an extensive collection of wildebeest carcasses accumulated by the same carnivore taxon in Southern Serengeti on a seasonal basin. This carnivore den is located at Olduvai Gorge (Tanzania), which is included in the short-grassland ecological unit of the Serengeti. A taphonomic study with special attention on carnivore bone modification patterns is being carried out. Preliminary taphonomic results have not shown features of a typical spotted hyaena or lion den (since no bone assemblage from a lion den has yet been documented), which is why both agents are explored in this work. The accumulating agent is known to be a carnivore because bones were collected in 2012 and new bones were found the following year at the site. This implies that an accumulating agent has been actively bringing carcasses into the den between the 2012 and 2013 research seasons.

The only prey present at the den is wildebeest, making the Olduvai den the first den documented with just one ungulate species. Furthermore, the anatomy of these wildebeest display a bone damage pattern that is consistent with that documented for carcasses modified by medium to large-sized felids (work in progress). Some of these diagnostic damage patterns include: lack of correlation between skeletal profiles and bone density, completeness of long bones, bone damage restricted to specific anatomic areas, such as those described by Domínguez-Rodrigo et al. [20] as characteristic of felid-induced bone damage patterns.

Analyses of age and sex classes of the wildebeest carcasses recovered at the den were carried out, as well as analyses of the seasonality of the accumulation. A multivariate analysis was applied and compared to previous results reported for lion and spotted hyaena kills [5,16]. The contradictions found between these recent data and previous ones shed some light on the differential sex and age class selection by predators in the Serengeti and the consequences for the dynamics of this ungulate population. This is of major importance for wildlife management research. The aim of this paper is the study of age and sex profiles which could help determine the agent of accumulation and the wildebeest population dynamics under which the den was created.

## Materials and Methods

**Fig 1:**
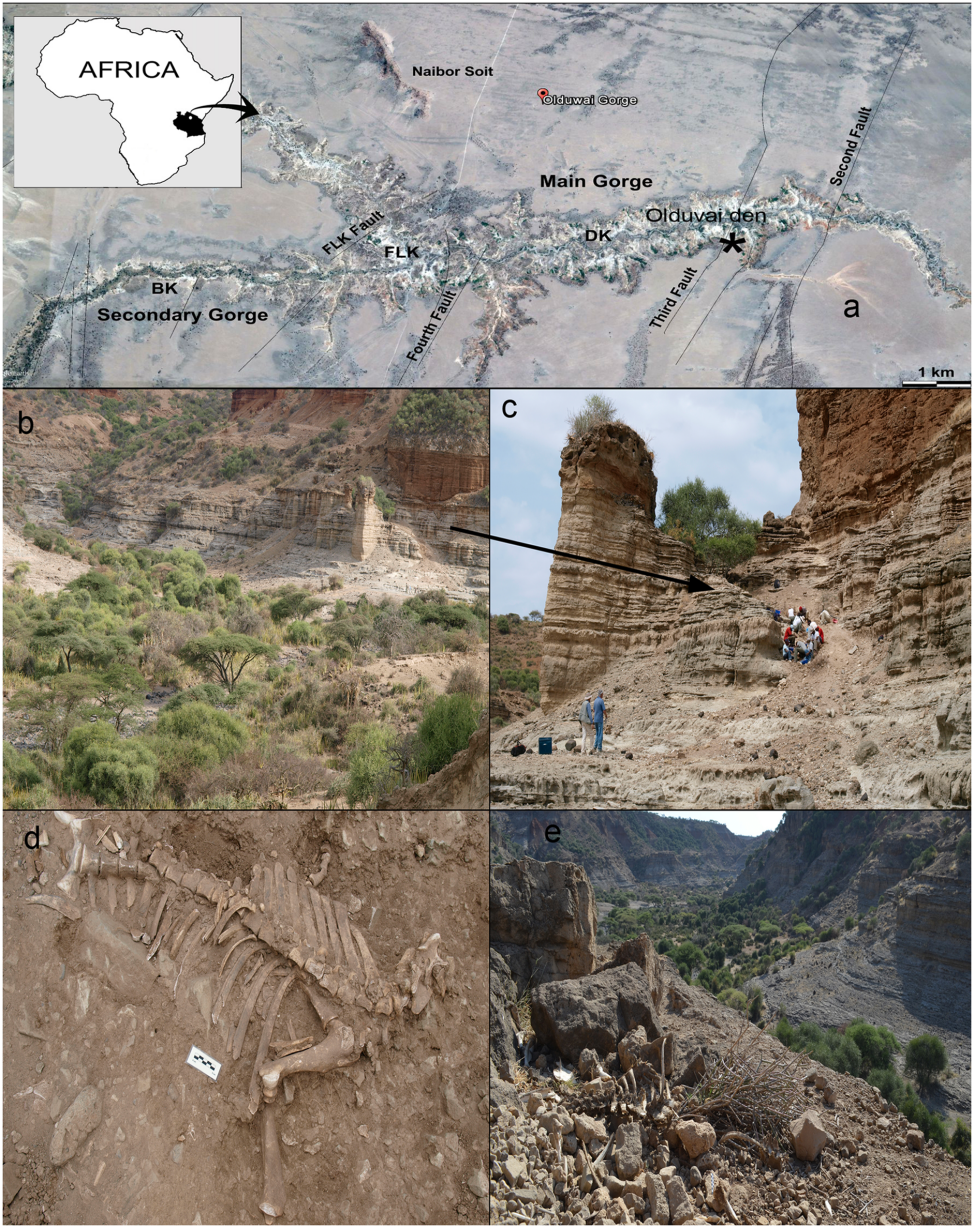
Location of the den and excavation work in the site. a) b) Location of the den (star) in Olduvai Gorge (Tanzania) c) Excavation work in the slope where the wildebeest bones were concentrated in 2012 d) One of the wildebeest carcasses recovered in 2012 e) One of the wildebeest carcasses recovered in 2013.

Olduvai Gorge is a valley at the western margin of the Eastern Rift Valley in northern Tanzania. The valley cuts the Serengeti Plain, which extends about 110 km northeast toward Lake Victoria [21]. A carnivore den was located close to the third fault of the gorge in 2012 (Olduvai Modern Den, Olduvai Gorge, Ngorongoro Conservation Area, Arusha, Tanzania) (Fig 1). It produced a large assemblage composed exclusively of wildebeest bones with conspicuous carnivore marks. The bones were concentrated on a slope situated close to a river course (Decimal latitude 35.392663; Decimal Longitude 2.985745). During the 2013 field season, upon finding new wildebeest bones at the site, we concluded that the den was active (Fig 1).

In 2012 we collected and excavated all the bones found at the slope of the den, along with bones dispersed at a distance up to a radius of 15 meters. In total, 4107 wildebeest bones were recovered. In 2013 we collected all the new bones found at the den: 426 wildebeest bones. This amounts to a sample of 4533 wildebeest bones in total. All necessary permits were obtained for the described study (Tanzania Commission for Science and Technology: COSTECH permit: 2014-174-ER-2006-115).

In order to determine how many individuals were present, we quantified skeletal part representation. Each specimen was identified to element, bone portion (in the case of limbs, these are proximal/distal epiphysis or diaphysis) and bone section (in the case of limbs, proximal/distal epiphysis, near-epiphysis shaft, and midshaft). The minimum number of elements (MNE) was calculated following Yravedra and Domínguez-Rodrigo [22]. This method gave an MNE estimate, taking account anatomical landmarks, the location of the shaft fragment on the complete bone as well as the size of individuals and relative age as reflected in cortical texture and body size. Minimal numbers of individuals (MNI) consisted of counting elements by siding paired bones and considering their age [23].

The relative age and sex of wildebeests is quite simple to determine in animals that are alive. Horn shape, body size and territorial behaviour are used to discriminate wildebeest sex classes [13–14]. These features are diagnostic to identify wildebeest age classes in the field [13–14]. Age and sex identification of a wildebeest carcass is possible as long as the skeleton preserves horns and teeth. In the present study, tooth eruption and wear pattern following criteria in Attwell [24] and Talbot and Talbot [13] were used as described by Sinclair and Arcese [16] to create five age classes: yearlings (1–2 years), young adults (2–4 years), mature adults (5–8 years), old (9–12 years) and very old (> 12 years) individuals. These age classes, determined by Sinclair and Arcese [16], are based on quantitative wear patterns of wildebeest teeth. This five-age class method is very accurate since it separates the crucial life stages of the wildebeest life history into yearlings and four stages from juveniles to adulthood, contrary to classical studies which separates age classes into three categories [17]. With this five-age method, changes in the recruitment rate of the ungulate population can be detected, as well as shifts in the diet of lion and spotted hyaena, since they prey on different adult classes [16]. The migratory wildebeest population of SNP has been monitored for 40 years (1958–1998) [5]. The population increased from 0.23 million to about 1.4 million from 1963 to 1977 (increase phase), stabilized from 1977 to 1993 (stable-phase) and declined during drought in 1993–94 [5]. Age class results were compared with those previously reported for lion and spotted hyena kill data from the increasing-stationary wildebeest phase (data from [16]) and decreasing wildebeest population phase (data from [5]) using multivariate statistics (see below). The specimen numbers studied were 9–44, 108–110, 117, 142–144, 149–150, 161, 173–175, 216–217, 251–253, 272 and 303.

Horn shape and its envelope are very accurate criteria in determining sex classes in wildebeest [14]. Unfortunately, horns do not always remain attached to the skull. Sexual dimorphism is shown in body size and thus in bone size [25]. In order to ascertain the sex of the individuals, six measurements were taken from adult metapodials using criteria from von Driesch [26] (Table 1). Metapodials are the best-represented complete adult bone in the sample and no measurements were taken of bones with a high degree of weathering. Studies which tested statistical methods for sex determination using bones biometrics are based on Principal Components Analysis [27]. Sex determination on six skulls recovered in 2013 was also carried out.

**Table 1:** Description of metapodials measurements for PCA analysis.

Variable	Description
Bd	Greatest breadth of the distal end
SD	Smallest breadth of the diaphysis
Bp	Greatest lateral-medial breadth of the proximal end
DD	Smallest depth of the diaphysis
GL	Greatest lenght
Anchura_2	Greatest cranio-caudal breadth of the proximal end

### Statistical method

Available age profiles from lion and spotted hyena kills are heterogeneous with samples from different authors varying greatly in size. To homogenize the available data and to make statistically valid assumptions, we bootstrapped 1000 times the original lion and hyena samples (including the den data) and then analyzed them with multivariate statistics. The approach adopted in our study was to create factors using the five-age class groups through Principal Component Analyses (PCA) and Canonical Variate Analyses (CVA). PCA and CVA produce factors that result from the reduction of dimensionality caused by multiple variables. PCA produce results that maximize sample variance. CVA focuses on data grouped into K classes by transforming original variables into canonical variables defined by square distances between the means of the groups obtained by Mahalanobis´s D^2^. CVA produces a higher degree of separation between the group means than PCA. In CVA, biplot axes are determined by the group means. PCA and CVA analyses were displayed with a 95% confidence interval ellipse. A PCA was also applied to fourteen left metacarpals and seventeen right metatarsals collected in 2012 for sex identification. Analyses and the bootstrapping procedure were performed and programmed in R (www.r-project.org). Graphic display of PCA and MDS bootstrapped tests were carried out with biplots using the R library “BiplotGUI”.

### Analysis of tooth histology for the study of the seasonal growth

**Fig 2:**
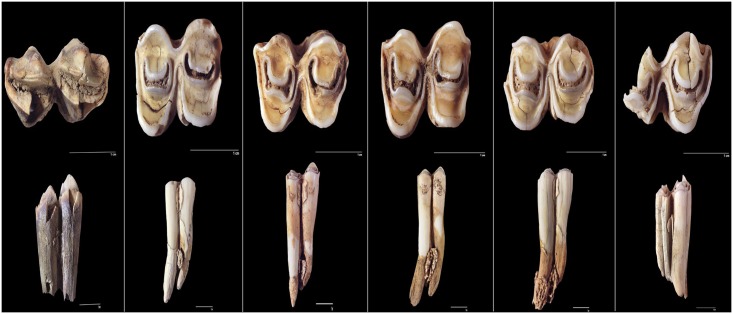
First molars used in histological analyses. First specimen is a juvenile. Adults are numbered from 506 to 510.

In the present work, we have carried out a pilot/exploratory study approaching the age and season at death of a small random sample of the wildebeest from the Olduvai den through the analysis of the histology of teeth [28]. For this study, we have analysed the first molars from specimens of different age (age inferred from the analysis of the eruption and wear of molars, see above), from five adults and one juvenile (Fig 2). Histological sections were obtained following standard protocols. First, each tooth was embedded in epoxy resin EpoFix (Struers) and then this block was cut longitudinally and in the bucco-lingual plane with a Struers Discoplan TS diamond saw. The cutting surface was ground and polished with a Buehler low-speed Isomet with SiC grinding papers (SiC-800, SiC-1200; Struers) to be fixed to a plexiglass slide using a cyanoacrylate adhesive. Subsequently, 200 μm-thick sections were cut using a Struers Discoplan TS diamond saw. Finally each histological section was ground and polished to a final thickness of 100μm with the use of different SiC grinding papers (SiC-800, SiC-1200; Struers). All necessary permits were obtained for this study, which complied with all relevant regulations (*Instituto de Evolución en África*). All histological sections obtained in this study are deposited in the Paleontology Collection of the *Instituto de Evolución en África* (Madrid, Spain) and are available to researchers. Thin sections were examined using an Olympus BX61 transmitted and polarized light microscope equipped with an Olympus DP71 digital camera (Servicio de Microscopia y Analisis de Imagen, Hospital Nacional de Parapléjicos, Toledo, Spain). The required images were merged and processed with Adobe Photoshop CS5 (Adobe Systems Inc).

To conduct our study, we have analysed the cementum and dentine regions of the first molars that are considered the best recording structures in ungulates [28]. However, in our sample, two specimens lack the dental root (where the cementum is located) and the remaining four specimens show the cementum highly altered. Therefore, we have focused our study on the structure of the dentin to infer the age and season at death. According to Klevezal ([28]; and citations therein), accentuated (dark) layers correspond to the growth layers formed annually coinciding with the unfavourable season (winter). By counting the number of dark layers within the dentin of the teeth we obtain the age-at-death for each specimen from this sample. The season-of-death was inferred considering the last dark layer (close to the pulp cavity).

## Results

### Age classes

**Fig 3:**
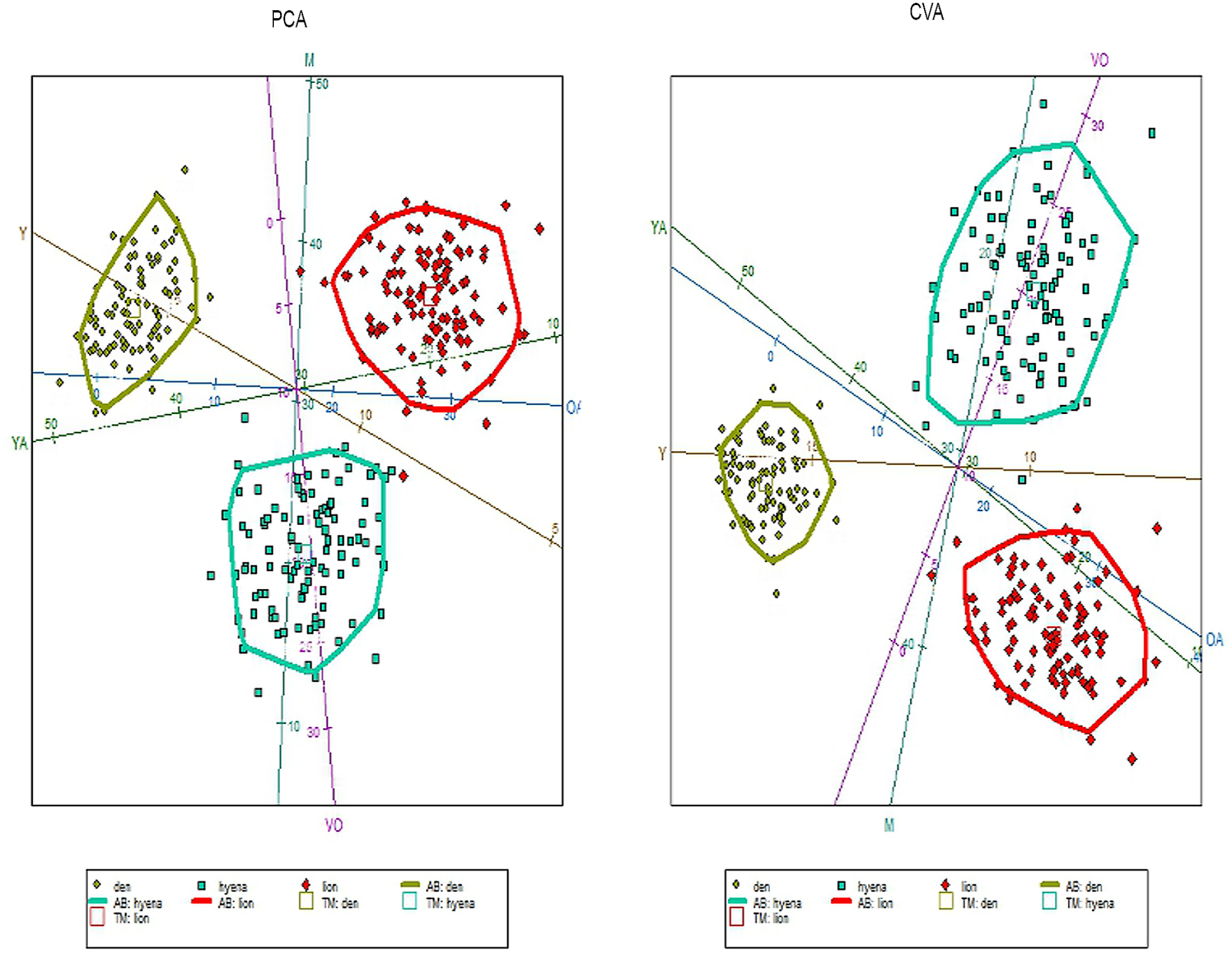
Biplot showing the distribution of the three comparative samples (lion kills, spotted hyena kills and Olduvai den) according to PCA (left) and CVA (right). Each sample also displays a 95% c.i. ellipse. Key: squares (hyenas), big diamonds (lions), small diamonds (den); Y, Yearlings; YA, Young Adults; M, Mature adults; OA, Old Adults, VO, Very Old. Data from spotted hyenas and lion kills (Mduma 1996; Sinclair & Arcese 1995).

**Fig 4:**
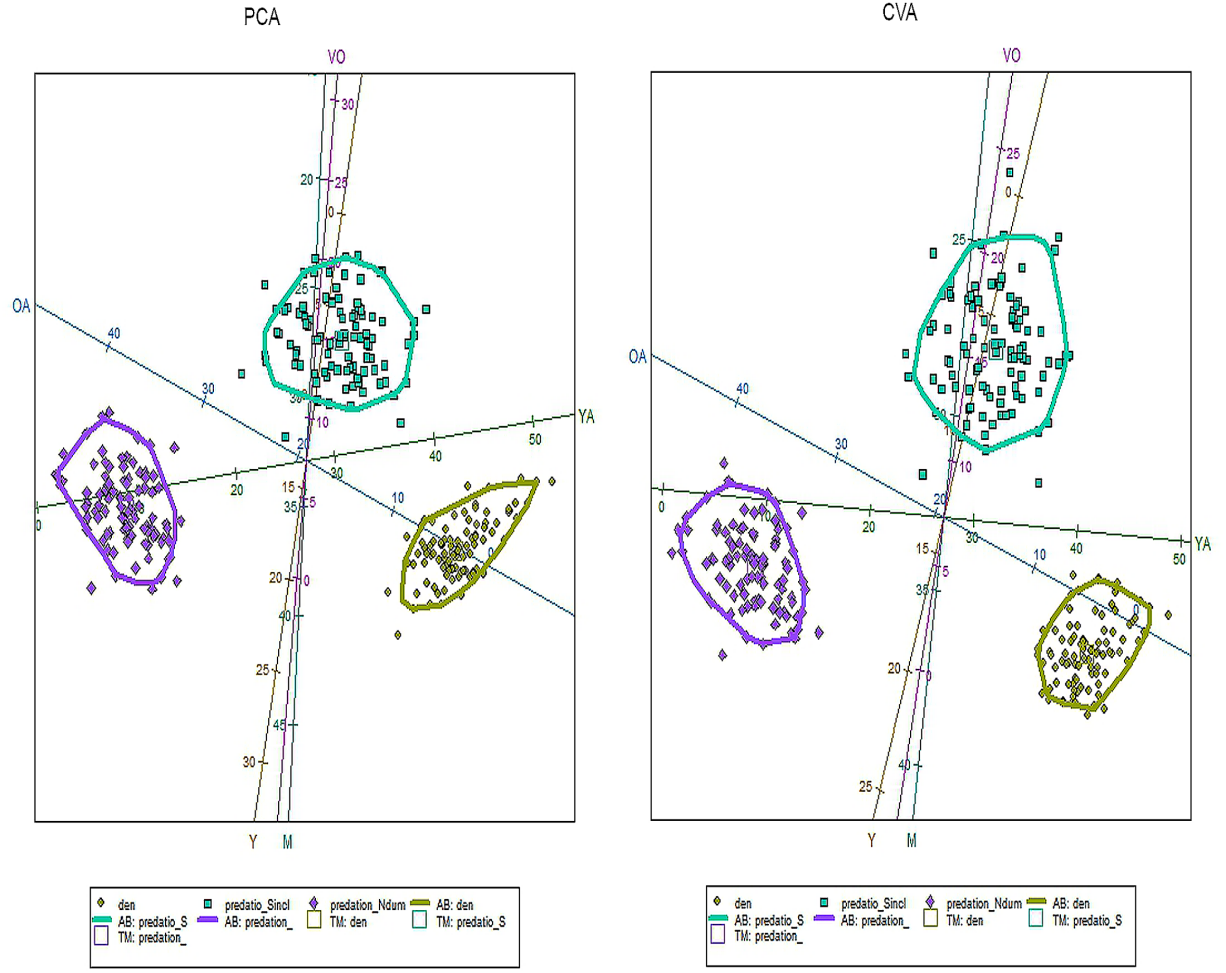
Biplot showing the distribution of the three comparative samples (total predation in the increasing-stationary phase (data from Sinclair & Arcese 1995), total predation in the decreasing phase (data from Mduma 1996) and Olduvai den) according to PCA (left) and CVA (right). Each sample also displays a 95% c.i. ellipse. Key: squares (total predation in the increasing-stationary phase), big diamonds (total predation in the decreasing phase), small diamonds (den); Y, Yearlings; YA, Young Adults; M, Mature adults; OA, Old Adults, VO, Very Old.

**Fig 5:**
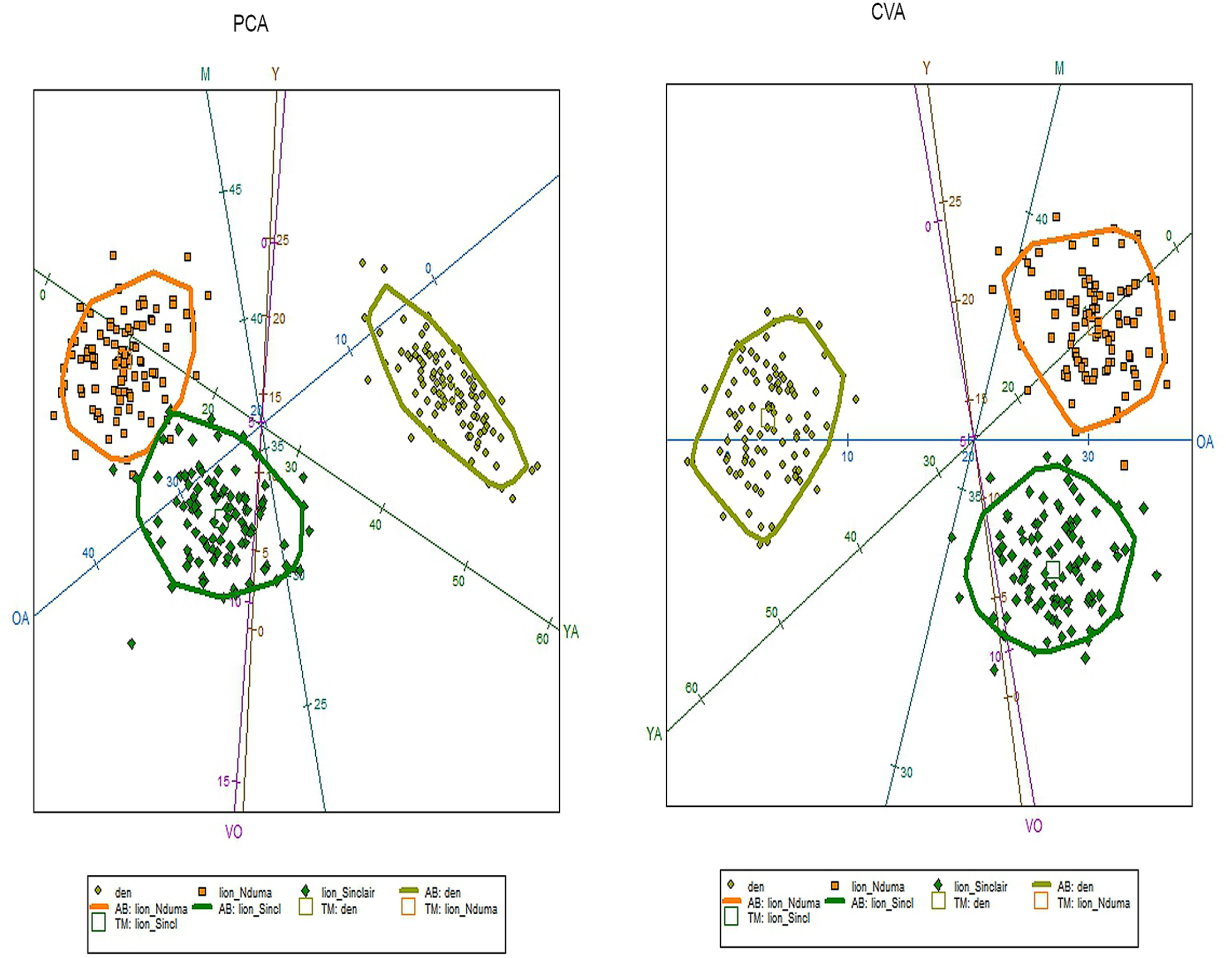
Biplot showing the distribution of the three comparative samples (lion kills in the increasing-stationary phase (data from Sinclair & Arcese 1995), lion kills in the decreasing phase (data from Mduma 1996) and Olduvai den) according to PCA (left) and CVA (right). Each sample also displays a 95% c.i. ellipse. Key: squares (lion kills in the decreasing phase), big diamonds (lion kills in the increasing-stationary phase), small diamonds (den); Y, Yearlings; YA, Young Adults; M, Mature adults; OA, Old Adults, VO, Very Old.

**Fig 6:**
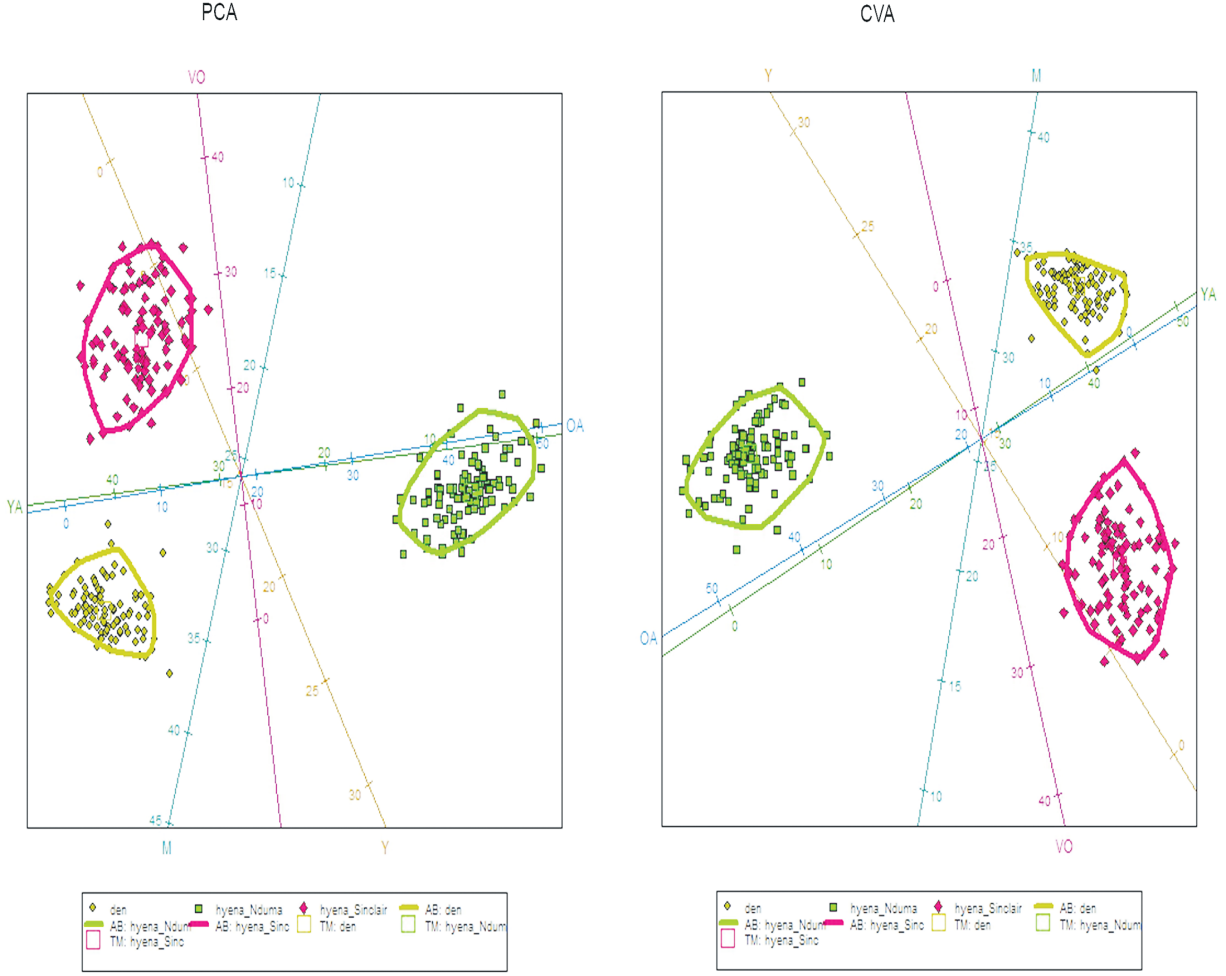
Biplot showing the distribution of the three comparative samples (spotted hyaena kills in the increasing-stationary phase (data from Sinclair & Arcese 1995), spotted hyaena kills in the decreasing phase (data from Mduma 1996) and Olduvai den) according to PCA (left) and CVA (right). Each sample also displays a 95% c.i. ellipse. Key: squares (spotted hyaena kills in the decreasing phase), big diamonds (spotted hyaena kills in the increasing-stationary phase), small diamonds (den); Y, Yearlings; YA, Young Adults; M, Mature adults; OA, Old Adults, VO, Very Old.

Fifty-five wildebeest were recovered at the den: 5 yearlings and 50 adults. Specific age class analyses on 29 individuals were carried out, because teeth usually are detached from mandibles and maxillae through dispersal mechanisms or weathering and for some adults only data from mandibles and maxillae provided accurate age estimates. Five individuals were yearlings, while the adult class was composed of thirteen young adults and eleven mature adults. No old or very old individuals were found. Skulls recovered in 2013, which comprised the carnivore accumulation created in just one year show two young adults and four mature adults. PCA and CVA results show no similarities between the Olduvai den results and the spotted hyaena and lion kills recovered from the increase-stationary phase along with the decrease phase of wildebeest population (Fig 3, Table A in S1 Table). The Olduvai den shows more yearling and young adult wildebeest, while the lion sample shows a higher presence of old adults. Hyenas prey on more very old individuals and fewer mature wildebeests. Furthermore, total predation (lion plus hyaena kills) are also dissimilar when compared in different years, depending on the wildebeest population dynamics (Fig 4, Table B in S1 Table). Total predation from the increase-stationary phase shows more very old individuals, while fewer very old and young adults were preyed during the decrease phase of wildebeest population. Furthermore, lion kill age profiles from the increase-stationary phase and from the decrease phase are not similar either (Table C in S1 Table). Fig 5 shows that during the increase-stationary phase more very old and young adult wildebeest and fewer mature adults and yearlings were preyed on by lions, whereas during the decrease phase, more mature and old adults and fewer young adults were killed by these felids. In addition, hyaena kills show differences depending on the growth rate of the wildebeest population. Hyena kills during the increase-stationary phase show more very old and young adult individuals and fewer yearlings, and during the decrease phase more old adults and yearlings and fewer young adults were killed (Fig 6, Table D in S1 Table).

### Sex ratio

**Fig 7:**
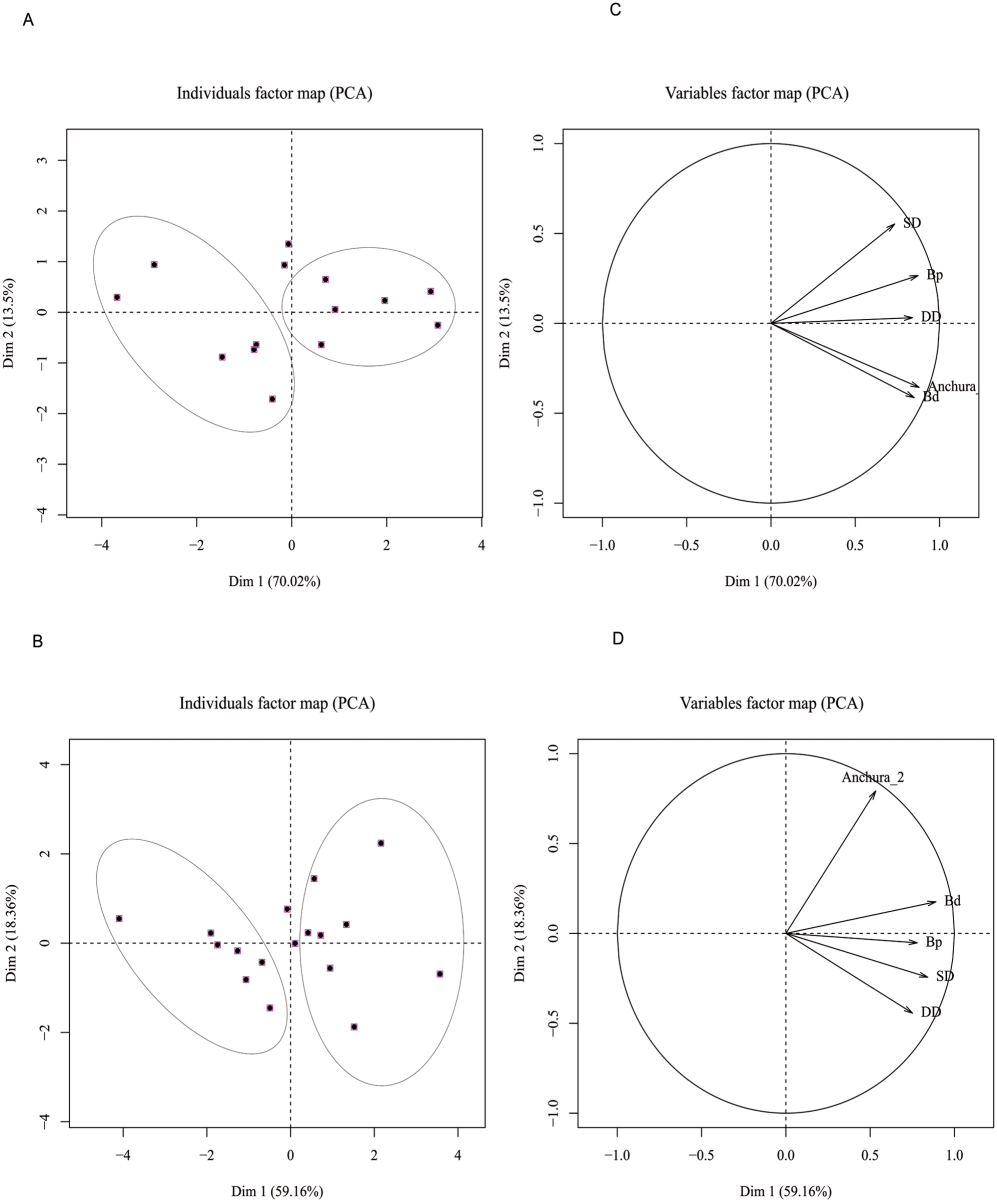
Spatial distribution of the Olduvai den specimens and biplot of the metric variables for metacarpals (A,C) and metatarsals (B,D) according to the PCA. Ellipses show groups of specimens identifying sexual dimorphism in the sample. Specimens outside ellipses remain ambiguous regarding sex identification.

PCA results show a sex ratio slightly skewed to females, in relation to metacarpals, or slightly skewed to males, in relation to metatarsals (Fig 7, table 2).

**Table 2:** Number of males and females according to the element used.

Element	Number of males	Number of females	Sex ratio
Skulls	2	4	1:2
Metacarpal	6	6	1:1
Metatarsal	7	8	~1:1

The variables that are most influential in the loading scores of the PCA for metacarpals are: the cranio-caudal breadth of the distal epiphysis and the maximum breadth of the proximal epiphysis for the first measurement. Shaft minimum breadth was the most important variable for the second measurement (Table E in S1 Table). Regarding metatarsals, the first measurement is accounted for the maximum and minimum breadths of the shaft and the second measurement is composed mostly of the cranio-caudal breadth of the distal epiphysis (Table F in S1 Table).

Sex classes determined by horn shape from the six skulls recovered in the 2013 field season shows a sex ratio skewed towards females (1:2): four individuals were female and two were males (Table 2).

### Seasonality

**Fig 8:**
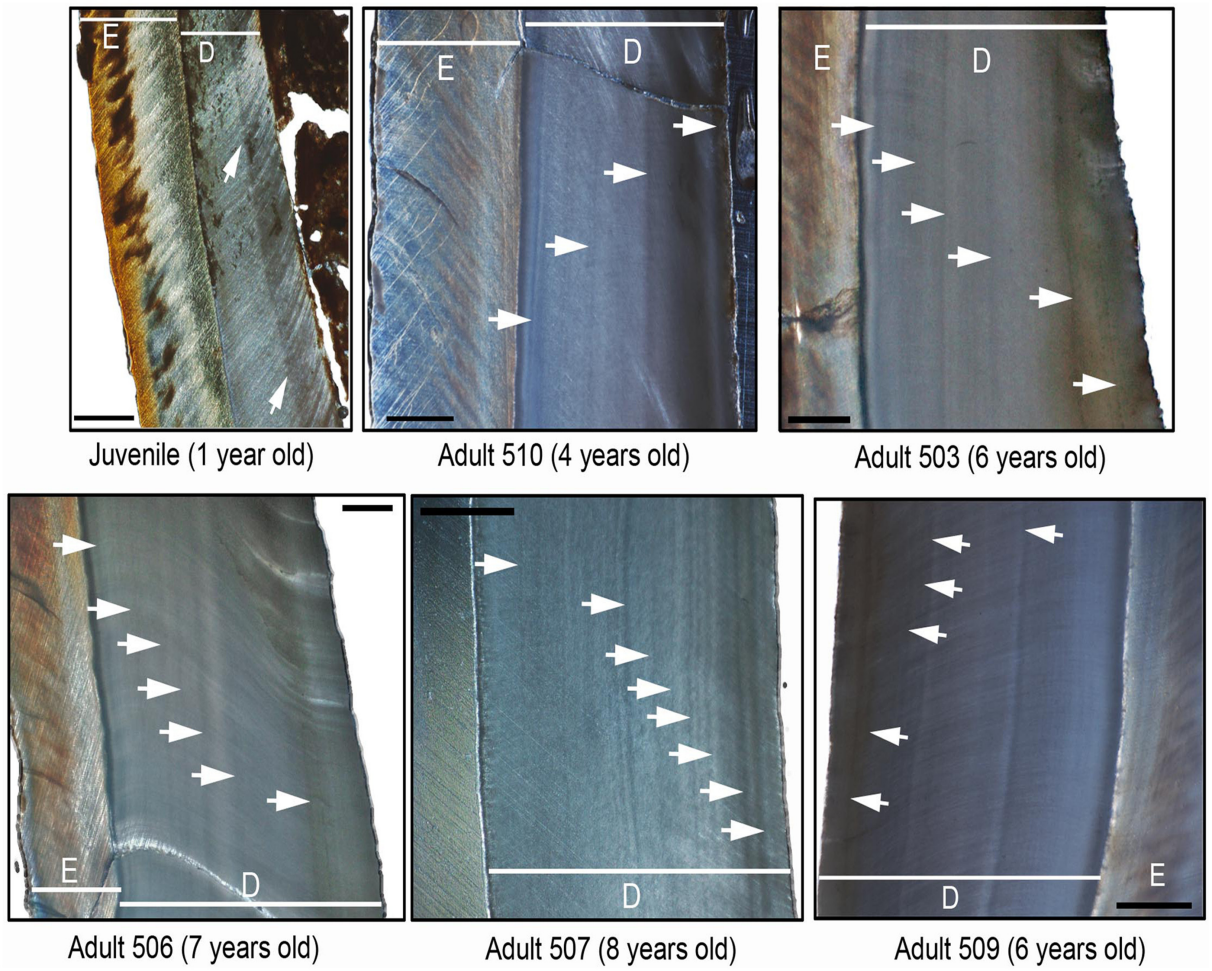
Detail of the dentine structure of the six first molars. This figure shows a close up of the enamel (E) and the dentin (D) regions. White arrows indicates the dark layers within the dentin which have been used to infer the age-at-death (between parentheses) and the season-of-death (dark layer is related to unfavourable season). Scalebar: μm.

In this work, we have inferred the season-of-death through the analysis of the histological structure of the dentin from a sample of first molars. All specimens show dark layers along the dentin, which allows us to infer the age-at-death of specimens by counting the number of layers ([28] and references therein, [29]; Fig 8). Thus, our sample consists of a one-year-old juvenile and five adult specimens from 4 to 8 years old. Concerning the season-of-death and considering that dark layers formed in unfavourable season [28–29], our results indicate that five specimens show a dark layer close to the wall of the pulp cavity indicating these individuals survived the last unfavourable season and resumed growth during the favourable season. In the adult specimen 510, a dark layer is observed on the edge of the cavity pulp wall that could be indicating that this specimen died in a unfavourable-favourable period. The favourable season in the Serengeti (the wet season) starts in November and December. The migratory wildebeest moves onto the Serengeti plains following this short rainy season. They remain on the plains as long as food is available. A major part of the wildebeest population moves eastward into the Serengeti Western Corridor once the plains dry out in May or June [5], when the unfavourable season (the dry season) begins.

## Discussion

The CVA and PCA results of our sample compared with previous data of spotted hyaena and lion kills [5,16] show that the Olduvai den is not correlated to either cursorial (spotted hyaena) or ambush (lion) predation (Fig 3). This implies that the referential data used as a comparative framework inaccurately describes the range of carnivore behaviours as conditioned by herbivore population dynamics. These previous models were made from data collected by Mduma [5] and Sinclair and Arcese [16]. These data describe predation patterns which are temporarily very limited and therefore do not incorporate all possible longitudinal behaviours occurring under different population dynamics. Data from Mduma [5] and Sinclair and Arcese [16] were taken during the complete year, since they did not find seasonal differences in their sample. In addition, their data come from different habitats comprised in three main vegetation zones (Serengeti short grassland plains, the central and western Acacia savannah and the northern Serengeti mixed Acacia and broad leafed woodlands) because neither Mduma [5] nor Sinclair and Arcese [16] found any statistical differences among these zones either. The seasonality results inferred from the histological analysis of the Olduvai wildebeest sample indicate that the bone accumulation at the Olduvai den occurred during the wet season. Considering that resumed growth after the rest period occurs shortly before death, we hypothesize that the season-of-death occurred during the early wet season, which means that predation occurred before the wildebeest breeding season [13–14].

Opportunistic foraging, frequently entailing seasonal diet changes, is often adopted by large carnivores in ecosystems where environmental resources vary by season [30]. Our results indicate a different age class selection by predators from those previously reported due to seasonality or habitat (or both), so both multivariate data of seasonality and habitat-type are necessary during the complete year to discriminate which predator was the responsible for the accumulation. Unfortunately, no data like this is available. Contrary to what Mduma et al. [31] point out, age distribution of kills by lions and spotted hyaenas differ strongly in their selection of age classes (Fig 3). Lions tended to kill proportionally more middle-aged animals (as in Mduma et al. [31]) and more old adults, compared with spotted hyaena kills (Fig 3). Spotted hyaena kills seem generally to be older than lion kills, as suggested in Sinclair and Arcese [16], because they prey on more very old individuals (Fig 3). Figs 5 and 6 show how spotted hyaena and lion predation is different depending on the growth rate of the wildebeest population. Age selection by predators varies depending on whether the wildebeest population is in the increase-stationary phase (data from [16]) or decreasing because of the drought in 1993 (data from [5]). This implies that major ecological changes can have an immediate impact on prey availability and selection by carnivores. Thus, changes in the age structure of the wildebeest population lead to differential age class selection by predators. This is clear since top-down processes do not regulate medium-size ungulate dynamics; these are rather influenced by bottom-up processes [32]. Lion kills, when wildebeest population growth rate is positive, are older than kills recorded when the population is decreasing (Fig 5). Lions select more very old and old individuals when the population is increasing and when intraspecific competition is lower in the ecosystem, which may be due to the higher occurrence of these age classes. When the wildebeest population is decreasing, these carnivores select more mature adults and yearlings and fewer young adults (Table 3). Conversely, spotted hyenas select more old adults and yearlings when the wildebeest population is decreasing, showing the classical attritional age class profile (Fig 6). When the wildebeest population is increasing, spotted hyaenas select more very old individuals and young adults (Table 3).

**Table 3:** Age classes selected by the lion and spotted hyaena depending on the wildebeest population phase.

	Increasing-stationary phase	Decreasing phase
Lion	+ Old adult +Very old adult	+ Mature adult + Yearling -Young adult
Spotted hyaena	+ Very old adult + Young adult	+ Old adult + Yearling

It is important to note that the five-age class method (instead of classical three-age class method) is crucial to detect changes in the diet of the lion and spotted hyaena. The five-age class method should be combined with multivariate statistics to detect changes in the diet of the different carnivores as wells as changes on the season or habitat type. Thereby, strong selection on age classes was first detected in both the lion and the spotted hyaena and showed different selection behaviours depending on the season and/or habitat and growth population rate. These data are crucial for a affective management of species and are needed to create good options for conservation. Furthermore, our methodological approach to estimate age cohorts is a valuable method to show the growth rate of the herbivore population through the study of age classes preyed by carnivores. Classical studies not based on wear patterns and with no common criteria to determine age classes [2,4] are of limited value for these goals.

The sex ratio in blue wildebeest population in the Serengeti is 1:1 [13–14]. Schaller [4] showed how lions hunt 2.3 males per each female in the wildebeest rut season in May in the Serengeti. In Kruger National Park (South Africa) Pienaar [3] pointed to wildebeest male differential predation by lions as well. In addition, Kruuk [2] showed hyenas hunt more wildebeest males than females in the Serengeti. Nevertheless, the metapodial data (Fig 7) and, especially, horn morphology results of our study show a sex ratio somewhat more balanced or skewed towards female wildebeests in the den, something which is not consistent with previous interpretations of male-dominated kills in lions and hyenas.

As noted above, the accumulation at the Olduvai den occurred in the early wet season. Kruuk [2] noted that in this period spotted hyena predation is skewed to female wildebeest because of their lower mobility in both the Serengeti and Ngorongoro ecosystems. This behaviour also occurs with lions in Kruger National Park (KNP), as shown by the sex classes of carcasses preyed in different seasons ([3], table 25, page 171). Wildebeest males are more highly represented in lion kills from March to the early dry season and wildebeest females from early wet season to January in the KNP [33]. Hence, the wildebeest population in this ecosystem is regulated by food availability, and predation entails additive mortality as soon as adult female wildebeests are killed by predators [33]. This could explain the skewed sex ratio in the den. Wildebeest carcasses preyed by lions and hyenas in the Serengeti recorded by Sinclair & Arcese [16] from 1977 to 1991 show an overall 1:1 sex ratio (Table 3, page 888). Therefore differential sex predation is not biased towards males but may rather vary seasonally and may be the mechanism that regulates the wildebeest sex ratio in the Serengeti. Differential predation skewed to female wildebeest is found in small reserves, too. Lehmann et al. [34] recorded more adult female wildebeest killed by lions than males in the Karongwe Game Reserve (Limpopo Province, South Africa) (Table 5, page 72). This result was also recorded in the Mala Mala Private Game Reserve (South Africa) ([35], Page 422, Appendix II). Thus, this mechanism may act in both enclosed reserves and large ecosystems such as KNP and SNP.

The rate of movement of female wildebeests in the Serengeti is lower in the wet season [10]. This could be a strategy related to the minimization of predation of less mobile newborns, or a comparatively higher exploitation of habitats of high quality [10], in order to obtain a good physical condition for the lactating phase [36]. The final months of gestation and the two months of lactation are the most nutritionally stressful for cows, therefore good grazing in December and January are critical [12,16,36]. Wildebeest maximum speed is very similar to that of lions [37] and may be slower for pregnant wildebeest. This could be the reason why female wildebeest are more vulnerable to predation in the wet season. Fertility rate is very high in blue wildebeest, being no lower than 80% of the female population [5]. Thus, it is very likely that female wildebeests found in the den were pregnant. Perinatal bones found in the den and histological results support this hypothesis, because the bone accumulation occurred before the breeding season.

Ecological models have shown that the population dynamics of sexually reproducing species are shaped by the mating system and the reproductive success of females [38–39]. The numbers of males would affect reproductive rate, equilibrium population densities and population stability [39–42]. Female mating rates can decrease with lower male numbers in the population, leading to a positive density dependence in the per-capita population rate known as the Allee Effect [38,43–45]. Skewed predation to males or females usually destabilizes populations, though male-biased predation of a limited polygynous system can lead to stable equilibrium predator-prey [38]. Arguably, female-biased sex ratio ensures successful male reproduction and assists in maintaining a polygynous mating system, while a ratio highly skewed towards females can lead to lower fertility rate and thus lower reproduction [46–47]. Serengeti´s non-migratory wildebeest population shows a female-biased sex ratio, longer birth period, lower fertility rate and higher calf mortality [48]. Skewed sex-ratio to females is explained by the differential male predation hypothesis and illegal hunting [48]. However, the Western Corridor, where this population is located, holds low spotted hyena and lion densities, and the harvest rate is small relative to the population size [10,31]. The female-biased sex ratio of this population may also be explained by its lacking differential sex predation in females in the wet season due to the absence of predators in this area. Skewed female sex ratio in this population can allow inexpert males to play an important role in the mating period, which can reduce the fertility rate and produce an asynchronous birth period [48]. Moreover, although adult pregnant female mortality may contribute negatively to population growth or stability, non-differential predation of sexes acted while the migratory wildebeest population was stabilized, in light of the results obtained by Sinclair & Arcese [16] from carcasses recorded from 1977 to 1991 in the Serengeti. Differential sex class predation by seasons in the migratory population may allow the maintenance of a population sex ratio close to 1:1. This enables sexual competition because, although males are mature by two years and have live spermatozoa in their tubules, most of them do not become sexually active until their fifth year because they are excluded from breeding by intense male competition [14,36]. Intense male competition enables a high fertility rate in the migratory population which is not lower than 80% [5] because exhausted bulls will stop competing and are quickly replaced by fresh bulls, meaning females are constantly harassed until they are bred [36,49]. The outcome is that, during the peak of the rut, 80% of reproductive active females are mated within a two to three week period [14,36], and this enables a synchronous birth period (which allow higher calf survival). Therefore, adult female deaths in the wet season are a trade-off for dynamic population stability.

The use of tooth histology and the biometrics of the bones (combined with horn morphology) have shown that the sex selection of predators depends on the season. These data have been crucial to create hypotheses about the natural history of the blue wildebeest and may be considered for further research on different ungulate species.

## Conclusions

A bone accumulation of 55 wildebeests was found inside Olduvai Gorge (Tanzania). Bone damage and the redundant accumulation of carcasses in between research seasons support a carnivore agency in the formation of this exceptional assemblage. Skeletons appear complete and with very slight bone modification, as is typical of felid consumption of carcasses [20]. The sex ratio is slightly biased toward females. The mortality profile is dominated by prime adult individuals. None of these characteristics are typical of either lions or spotted hyenas (potential predators of wildebeest) as previously documented. However, previous referential age profile frameworks were elaborated on data collected for a limited amount of time, and did not sample all the range of potential behaviors exhibited by solitary and reproductive lions or prides during specific seasons. A preliminary analysis of seasonality shows that the accumulation of the den took place at the beginning of the wet season, prior to the wildebeest birth season. Monitoring of the den during the dry season also supports this preliminary reconstruction since carcasses were not accumulated during this period. During the early wet season, preference of lions for females over males has been documented [3,33].

A separate taphonomic study shows that felids and no spotted hyenas were responsible for the bone accumulation at the den (work in progress). The attribution of this accumulation of wildebeest to lions and no leopards (more eclectic in the taxonomic diversity of its prey and regularly transporting carcasses under 120 kg) has major consequences for the interpretation of the behavior of prehistoric carnivores. First, this would be the first well-documented bone accumulation made by lions. Secondly, until now a prime-adult assemblage had been the interpreted as the landmark of a human agency [17, 50–51]. The den assemblage shows that at least one carnivore type could also accumulate prime adult carcasses. This assemblage would force the revision of current referential frameworks to understand age and sex profiles for prehistoric carnivores. It would also influence the revision of current methods of estimating age profiles for wildlife management, suggesting the use of a minimum of five-age classes. The present study shows that predator-prey relationships can only be understood longitudinally over extended periods of time and always in relation to changing prey population dynamics in relation to modifications in the ecosystem. Future studies on lions and spotted hyenas focused on these premises from a larger set of ecosystems will provide evidence of a wider range of behaviours. These should eventually contribute to our understanding of wildlife populations and their management.

## Supporting Information

S1 Table Loading scores of the correlation values of the PCA and CVA analysis. (DOCX)
