# Supplementary material for: Differential Predation by Age and Sex Classes in Blue Wildebeest in Serengeti: Study of a Modern Carnivore Den in Olduvai Gorge (Tanzania)
Source: PLoS One. 2015 May 27;10(5):e0125944. doi: 10.1371/journal.pone.0125944 (PMC4446270; doi:10.1371/journal.pone.0125944)
Supplement: S1 Table — (DOCX) [file pone.0125944.s001.docx]

**S1 Tables: Loading scores of the correlation values of the PCA and CVA analysis.**

Table A. Loading scores of the correlation values of the PCA and CVA for lion kills (data from Mduma 1996 plus Sinclair & Arcese 1995), spotted hyaena kills (data from Mduma 1996 plus Sinclair & Arcese 1995) and Olduvai den. PCA Variance= 89.7. CVA Variance=92.6.

|  | Dim.1 PCA |  | Dim.1 CVA |
| --- | --- | --- | --- |
| Old Adult | 0.95 | Old Adult | -0.68 |
| Young Adult | 0.91 | Young Adult | 0.62 |
| Yearling | 0.36 | Yearling | 0.17 |
| Very Old | 0.04 | Very Old | -0.19 |
| Mature Adult | 0.0003 | Mature Adult | 0.09 |
|  | Dim.2 PCA |  | Dim.2 CVA |
| Mature Adult | 0.93 | Mature Adult | -0.70 |
| Very Old | 0.84 | Very Old | 0.56 |
| Yearling | 0.03 | Yearling | -0.01 |
| Young Adult | 0.02 | Young Adult | 0.20 |
| Old Adult | 0.00002 | Old Adult | -0.06 |

Table B. Loading scores of the correlation values of the PCA and CVA for total predation (lion plus spotted hyaena kills) from the increasing-stationary phase (data from Sinclair & Arcese 1995), the decreasing phase (data from Mduma 1996) and Olduvai den. PCA Variance= 92.9. CVA Variance= 97.7.

|  | Dim.1 PCA |  | Dim.1 CVA |
| --- | --- | --- | --- |
| Young Adult | 0.97 | Old Adult | 0.99 |
| Old Adult | 0.94 | Young Adult | 0.97 |
| Yearling | 0.06 | Yearling | 0.02 |
| Very Old | 0.05 | Very Old | 0.008 |
| Mature Adult | 0.02 | Mature Adult | 0.002 |
|  | Dim.2 PCA |  | Dim.2 CVA |
| Very Old | 0.83 | Mature Adult | 0.99 |
| Yearling | 0.76 | Very Old | 0.99 |
| Mature Adult | 0.59 | Yearling | 0.98 |
| Old Adult | 0.04 | Young Adult | 0.03 |
| Young Adult | 0.004 | Old Adult | 0.0002 |

Table C. Loading scores of the correlation values of the PCA and CVA for lion kills from the increasing-stationary phase (data from Sinclair & Arcese 1995), the decreasing phase (data from Mduma 1996) and Olduvai den. PCA Variance= 91.7. CVA Variance= 97.4.

|  | Dim.1 PCA |  | Dim.1 CVA |
| --- | --- | --- | --- |
| Young Adult | 0.95 | Old Adult | 0.99 |
| Old Adult | 0.92 | Young Adult | 0.93 |
| Mature Adult | 0.13 | Mature Adult | 0.44 |
| Very Old | 0.03 | Very Old | 0.09 |
| Yearling | 0.02 | Yearling | 0.07 |
|  | Dim.2 PCA |  | Dim.2 CVA |
| Yearling | 0.82 | Yearling | 0.94 |
| Very Old | 0.45 | Very Old | 0.91 |
| Mature Adult | 0.22 | Mature Adult | 0.56 |
| Old Adult | 0.06 | Young Adult | 0.07 |
| Young Adult | 0.04 | Old Adult | 0.009 |

Table D. Loading scores of the correlation values of the PCA and CVA for spotted hyaena kills from the increasing-stationary phase (data from Sinclair & Arcese 1995), the decreasing phase (data from Mduma 1996) and Olduvai den. PCA Variance= 94.8. CVA Variance= 96.2.

|  | Dim.1 PCA |  | Dim.1 CVA |
| --- | --- | --- | --- |
| Old Adult | 0.98 | Young Adult | 0.99 |
| Young Adult | 0.97 | Old Adult | 0.99 |
| Yearling | 0.42 | Yearling | 0.57 |
| Mature Adult | 0.18 | Mature Adult | 0.22 |
| Very Old | 0.09 | Very Old | 0.13 |
|  | Dim.2 PCA |  | Dim.2 CVA |
| Very Old | 0.88 | Very Old | 0.87 |
| Mature Adult | 0.62 | Mature Adult | 0.78 |
| Yearling | 0.33 | Yearling | 0.43 |
| Old Adult | 0.004 | Old Adult | 0.009 |
| Young Adult | 0.002 | Young Adult | 0.006 |

Table E. PCA loading scores of the correlation values of metric variables and their significance for metacarpals. See key to variables in Table 1.

| Dim.1 | correlation | p.value |
| --- | --- | --- |
| Anchura_2 | 0.88 | 3.465182e-05 |
| Bp | 0.87 | 4.595236e-05 |
| Bd | 0.85 | 1.184963e-04 |
| DD | 0.84 | 1.709721e-04 |
| GL | 0.76 | 1.702163e-03 |
| SD | 0.73 | 2.802401e-03 |
| Dim.2 | correlation | p.value |
| SD | 0.55 | 0.04029872 |

Table F. PCA loading scores of the correlation values of metric variables and their significance for metatarsals. See key to variables in Table 1.

| Dim.1 | correlation | p.value |
| --- | --- | --- |
| Bd | 0.89 | 1.709712e-06 |
| SD | 0.84 | 2.247273e-05 |
| Bp | 0.78 | 2.243850e-04 |
| DD | 0.75 | 5.047380e-04 |
| GL | 0.56 | 1.940374e-02 |
| Anchura_2 | 0.53 | 2.747219e-02 |
| Dim.2 | correlation | p.value |
| Anchura_2 | 0.79 | 0.0001473453 |
